# Development of hepatic steatosis in male and female mule ducks after respective force-feeding programs

**DOI:** 10.3389/fphys.2024.1392968

**Published:** 2024-06-21

**Authors:** Elham Atallah, Sabrina Trehiou, Valérie Alquier-Bacquie, Frédéric Lasserre, Julien Arroyo, Caroline Molette, Hervé Remignon

**Affiliations:** ^1^ Toxalim (Research Centre in Food Toxicology), INRAE, ENVT, UPS, Université de Toulouse, Toulouse, France; ^2^ Euralis Gastronomie, Maubourguet, France; ^3^ INP-ENSAT, Université de Toulouse, Castanet-Tolosan, France

**Keywords:** fatty liver, cellular stress, mule duck, sexual dimorphism, liver steatosis

## Abstract

Male and female mule ducks were subjected to a force-feeding diet to induce liver steatosis as it is generally done only with male ducks for the production of foie gras. The different biochemical measurements indicated that the course of hepatic steatosis development was present in both sexes and associated with a huge increase in liver weight mainly due to the synthesis and accumulation of lipids in hepatocytes. In livers of male and female ducks, this lipid accumulation was associated with oxidative stress and hypoxia. However, certain specific modifications (kinetics of lipid droplet development and hepatic inflammation) indicate that female ducks may tolerate force-feeding less well, at least at the hepatic level. This is in contradiction with what is generally reported concerning hepatic steatosis induced by dietary disturbances in mammals but could be explained by the very specific conditions imposed by force-feeding. Despite this, force-feeding female ducks seems entirely feasible, provided that the final quality of the product is as good as that of the male ducks, which will remain to be demonstrated in future studies.

## Introduction

Foie gras is a typically renowned French dish and is part of French gastronomy included on the UNESCO’s list of intangible heritage of humanity since 2010. It comes from the force-feeding of waterfowls, mainly ducks. Force-feeding is carried out over a short period (around 10 days) but includes very large quantities of a very caloric meal, almost exclusively composed of corn, which quickly induces hepatic steatosis. Indeed, under these conditions, the liver of birds quickly directs its metabolism toward a very intense production of lipids which are only partially exported and therefore accumulated in the hepatocytes (Heraut et al., 2010; Bax et al., 2012; Bonnefont et al., 2019; Lo et al., 2020). The result of the force-feeding of male ducks is similar, at least at the hepatic level, to what is also described in humans for non-alcoholic fatty liver disease (NAFLD) and/or non-alcoholic steatohepatitis (NASH) (Raza et al., 2021).

For the production of duck foie gras, only male ducks are generally used due to the sexual dimorphism that exists in birds (Brun et al., 2015): male ducks are larger than female ducks and are therefore easier to force-feed. Male ducks are also said to be calmer than female ducks and therefore easier to handle during periods of rearing and force-feeding (Basso et al., 2014). In addition, a significant portion (20%–40%) of female ducks has a liver with a large and superficial venous network, which ultimately makes the product unattractive to consumers (Marie-Etancelin et al., 2015). The origin of this visual defect is not yet documented even if it seems to be of genetic origin and independent of the force-feeding operations themselves.

In recent years, global poultry production has been faced with numerous successive avian influenza crises which have had a significant impact on production and breeding flocks (Spackman, 2020). Among all the consequences of this massive destruction of several million wild and domestic birds, the reduced availability of male ducks for the production of foie gras has sparked interest in the possible substitute use of female ducks. However, until now, no specific information has been published on possible differences in the development of steatotic liver in male or female ducks, while it has been widely documented in mammals of various species (Lornardo et al., 2019; Lefebvre and Staels, 2021).

The aim of this article was therefore to describe separately the evolution of hepatic steatosis in male and female ducks subjected to two different force-feeding programs adapted to their original body sexual dimorphism.

## Material and methods

### Animals and liver sampling

A flock of approximately 1,000, 50% males + 50% females, mule ducks (*Caïrina moschata* x *Anas platyrhynchos*) was reared, on the same farm, for 12 weeks, from hatching, according to usual commercial rules. At 12 weeks of age, 12 birds from both sexes (live weights similar to the average live weight of their respective breeding flocks) were randomly selected and slaughtered to constitute the group of ducks (0 meal) before the start of force-feeding. At each stage of sampling, venous livers (exclusively seen in female ducks at a percentage of 20%–30%) were discarded to retain only the non-venous livers from both sexes. The remaining birds were subjected to two different gender-specific force-feeding programs of 21 meals (twice daily for 10 days) using moistened corn flour (97.5% corn) on independent farms and poultry houses. At the start of the force-feeding period, male and female ducks received an amount of 225 g of dry flour/meal, which was gradually, but differently, increased in both sexes to reach a final value of 510 g and 480 g for the last meal in male and female ducks, respectively. In total, during the entire force-feeding period, male and female ducks were forced to ingest different quantities of feed (8.4 kg and 8.0 kg, respectively) depending on their different body sizes. Therefore, this experiment is not a true comparison of the effect of a given force-feeding program on the development of fatty livers in male and female ducks. Rather, it is an analysis of the development of hepatic steatosis in male and female ducks in response to two force-feeding programs best suited to their respective sizes. After 8 and 16 meals of force-feeding, 12 animals of both sexes were randomly selected (in the same manner as described above for ducks at 0 meals) and slaughtered to constitute the 8- (8 meals of force-feeding) and 16-meal groups (16 meals of force-feeding). The remaining birds continued the force-feeding programs for five additional meals. At this time, all the birds were slaughtered, and 24 of them (12 males and 12 females) were selected to constitute the 21-meal group (end of the force-feeding period). At this final stage, because the data provided by the slaughterhouse allowed it, the selection of livers after 21 force-feeding meals was carried out according to the average weights of the livers observed for each sex in the remaining birds (mean ± SD = 566 g ± 93 and 517 g ± 92 for males and females, respectively). At each stage (0, 8, 16, and 21 meals), the ducks were slaughtered 12 h h after the last meal in a commercial slaughterhouse according to its standardized slaughter operations (electronarcosis, bleeding, scalding, and plucking). At the end of the slaughtering line, 20 min *post mortem*, the livers were harvested and weighed. Then, 50 g of tissue was collected from the median lobe and directly frozen in liquid nitrogen before storage at -80°C. Another piece of the liver was also collected from the same location and stored in paraformaldehyde (4%) buffer for histological observations.

All biochemical measures were performed in duplicate after grinding the tissues in liquid nitrogen.

### Gross biochemical composition of livers

The dry matter (DM) content was determined by drying the ground liver in an oven at 105°C for 24 h. The total lipid content was measured according to Folch et al. (1957) after extraction with chloroform:methanol (2:1). The total protein content was determined according to the procedure described by the manufacturer (Pierce™ BCA Protein Assay Kit) after an extraction with a phosphate-buffered saline solution. The hydroxyproline (OH-Pro) content was determined according to Woessner (1961) on the delipidated and dry residue obtained after the extraction of the total lipids.

### Oxidative status

GSH/GSSG analysis: The reduced glutathione/oxidized glutathione (GSH/GSSG) ratio was determined according to the protocol described by the manufacturer (catalog #: 239709, Abcam, Cambridge, United Kingdom).

The activities of the enzymes superoxide dismutase (SOD, catalog #: 19160, Sigma, St Louis, MO, United States) and catalase (Cat, catalog #: KB03012, BioQuoChem, Llanera-Asturias, Spain) were determined according to the procedures described by the manufacturers.

The results are expressed in U/mg of proteins.

### ELISA tests

The contents of hypoxia-inducible factor 1 alpha (HIF1α) and hypoxia-inducible factor 2 alpha (HIF2α) were determined with ELISA tests on the proteins extracted from livers by using assay kits from MyBioSource (San Diego, CA, United States) according to the manufacturer’s protocols.

Results are expressed in pg/mg of proteins.

### Histology

Paraformaldehyde-fixed and paraffin-embedded liver tissue sections (3 µm) were stained with hematoxylin and eosin (H&E) for histopathological analysis. The stained liver sections were analyzed (magnification ×100) blindly according to a score ranging from 0 = no visible lipid droplets, 1 = only small lipid droplets, 2 = majority of small lipid droplets, 3 = majority of large lipid droplets, to 4 = almost only large lipid droplets in hepatocytes. Mean score values were obtained from three independent, trained observers.

### Gene expression

Total cellular RNA was extracted from liver samples using the TRI reagent (Molecular Research Center Inc., Cincinnati, Ohio, United States). RNA was quantified using a NanoPhotometer (N60, Implen). Total RNA samples (2 µg) were reverse-transcribed using the High-capacity cDNA Reverse Transcription Kit (Applied Biosystems, Foster City, California, United States) for real-time quantitative polymerase chain reaction (qPCR) analyses. Primers were designed in two consecutive exons to avoid amplification of genomic DNA using PrimerQuest^™^ Tool (Integrated DNA Technologies, Coralville, Iowa, USA), and primers for SYBR Green assays are presented in Supplementary Table S1. Amplifications were performed on the AriaMx Real-time PCR System (Agilent, Santa Clara, California, United States). RT-qPCR data were normalized to the level of the GAPDH (glyceraldehyde-3 phosphate dehydrogenase) messenger RNA (mRNA) and analyzed by LinRegPCR (v2021.2). This program determines the PCR efficiency per sample and accounts for it in a linear regression approach to correct the cycle threshold value for mRNA level quantification. The initial concentration (N0) for each sample is calculated using N0 = threshold/(Effmean x Cq), with Effmean representing the mean PCR efficiency and Cq representing the quantification cycle.

For each analyzed transcript, the value at the beginning of the force-feeding period was set to 1, in each sex, to facilitate the comparisons.

### Statistics

Statistical analyses were performed with SAS software, version 9.4, of the SAS System for Windows. Analysis of variance were performed with the general linear model (Proc GLM) completed with the Student–Newman–Keuls *post hoc* test to compare the means obtained after each meals in each of the two groups (sexes) independently. Where necessary, to satisfy normality and homoscedasticity conditions, variables were transformed before analysis (Log_2_ for RT-PCR and OH-Pro analysis). The percentages of the respective scores from the histological analysis were compared independently for each gender according to Fischer’s exact test. Values are expressed as the means ± standard deviation (SD). We set the significant level at *p* < 0.05.

## Results and discussion

Throughout the experiment, total mortality (number of animals dead at the end of the rearing–feeding periods/number of animals at the beginning) was less than 1.5% in both sexes, indicating that almost all ducks were able to withstand the rearing conditions imposed. As expected, the force-feeding programs induced a huge increase in liver weight (Table 1). If, at the beginning of the force-feeding period, the weight of the liver of all the birds was approximately 125 g, nevertheless with a slightly higher value (+2.3%) in male ducks, after 21 meals, the weight of the livers increased by 4.51 and 4.23 in male and female ducks, respectively. These values were identical to those expected, at least in male ducks, for which references under close force-feeding conditions are available (Gabarrou et al., 1996, 695 g for 12.5 d of force-feeding; Bax et al., 2012, 660 g for 12 d of force-feeding; and Bonnefont et al., 2019, 600 g for 10 d of force-feeding). In both sexes, lipid levels increased (from 5.0% to 5.3% at meal 0 in males and females, respectively, to 58.3% and 60.5% at meal 21), while protein levels decreased throughout the overfeeding period. The percentage of lipids was then multiplied by more than 11 between 0 and 21 forced meals in both sexes. This indicates a spectacular accumulation of lipids and, therefore, the development of clear hepatic steatosis. This is due to the rapid transformation by the liver of the large quantities of carbohydrates provided by the successive corn meals (containing 62% starch) imposed by the force-feeding programs. It must therefore be concluded that male and female ducks both retain this capacity to transform sugars from the diet into lipids, which accumulate in the liver during the force-feeding period. This ability to accumulate lipids under these particular conditions has already been described in several experiments conducted with male ducks (Herault et al., 2010; Lo et al., 2020; Pioche et al., 2020; Tavernier et al., 2020). The liver’s hydroxyproline (OH-Pro) content reflects its capacity to develop connective tissues, and this indicator has previously been used (Arai et al., 2022; Montefusco et al., 2022) to illustrate the shift from the simple steatosis associated with non-alcoholic fatty liver disease (NAFLD) to non-alcoholic steatohepatitis (NASH). In mice, an increase in the liver’s OH-Pro content was reported by Matsumoto et al. (2013) and Hartimath et al. (2019) and considered a sign of the development of fibrosis characteristic of the NASH condition. In the present experiment, we report a significant increase (+179% and +236%, *p* < 0.05, in males and females, respectively) in the OH-Pro contents of livers only between 16 and 21 meals of force-feeding. This indicates that only at the very end of the force-feeding period, a fibrogenesis process could take place in both sexes. This is, however, contradictory to what had previously been reported in male ducks by Remignon and Burgue (2023), who had not observed such an increase. However, this last observation was the first published in force-fed ducks, and it is therefore difficult to recognize whether the current results are atypical or not, even if the presence of hepatic inflammation attested by an increase in its fibrogenesis seems rather logical in light of what is generally observed in severe cases of hepatitis steatosis in mammals (Schuppan et al., 2018; Schwabe et al., 2020).

**TABLE 1:** Chemical composition of the livers according to the sex of the mule ducks and the number of meals during the force-feeding period (*n* = 12/sex/stage). Values are the means ± SD.

Gender	Parameter	0 meal	8 meals	16 meals	21 meals	P^3^ <
Male	Liver weight (g)	127^a^ ± 15	276^b^ ± 25	426^c^ ± 65	573^d^ ± 14	0.0001
	Humidity^1^	66.9^a^ ± 0.8	50.7^b^ ± 2.6	36.1^c^ ± 2.3	31.7^d^ ± 2.3	0.0001
	Lipids^1^	5.0^d^ ± 0.8	31.6^c^ ± 3.0	50.7^b^ ± 2.2	58.3^a^ ± 4.9	0.0001
	Proteins^1^	7.9^a^ ± 1.2	5.9^b^ ± 0.7	4.4^c^ ± 0.9	3.4^d^ ± 0.3	0.0001
	OH-Pro^2^	160^b^ ± 22	104^b^ ± 8	144^b^ ± 19	258^a^ ± 41	0,0016
Female	Liver weight (g)	124^a^ ± 21	257^b^ ± 25	422^c^ ± 56	525^d^ ± 17	0.0001
	Humidity^1^	67.1^a^ ± 1.0	48.3^b^ ± 2.0	37.0^c^ ± 2.2	28.8^d^ ± 1.9	0.0001
	Lipids^1^	5.3^d^ ± 1.3	35.3^c^ ± 4.7	51.0^b^ ± 6.3	60.5^a^ ± 3.5	0.0001
	Proteins^1^	9.4^a^ ± 2.9	7.2^b^ ± 0.9	5.0^c^ ± 1.1	3.1^d^ ± 0.4	0.0001
	OH-Pro^2^	189^b^ ± 32	139^b^ ± 11	177^b^ ± 16	417^a^ ± 60	0.0001

Means with the same superscripts are not different (*p* < 0.05). Within a line, mean values with different superscripts are different (*p* < 0.05).

^1^: % of raw liver.

^2^: Hydroxyproline (OH-Pro) content in mg/g of delipidated and dry liver.

^3^: *p*-values are related to the effect of the number of meals.

**FIGURE 1:**
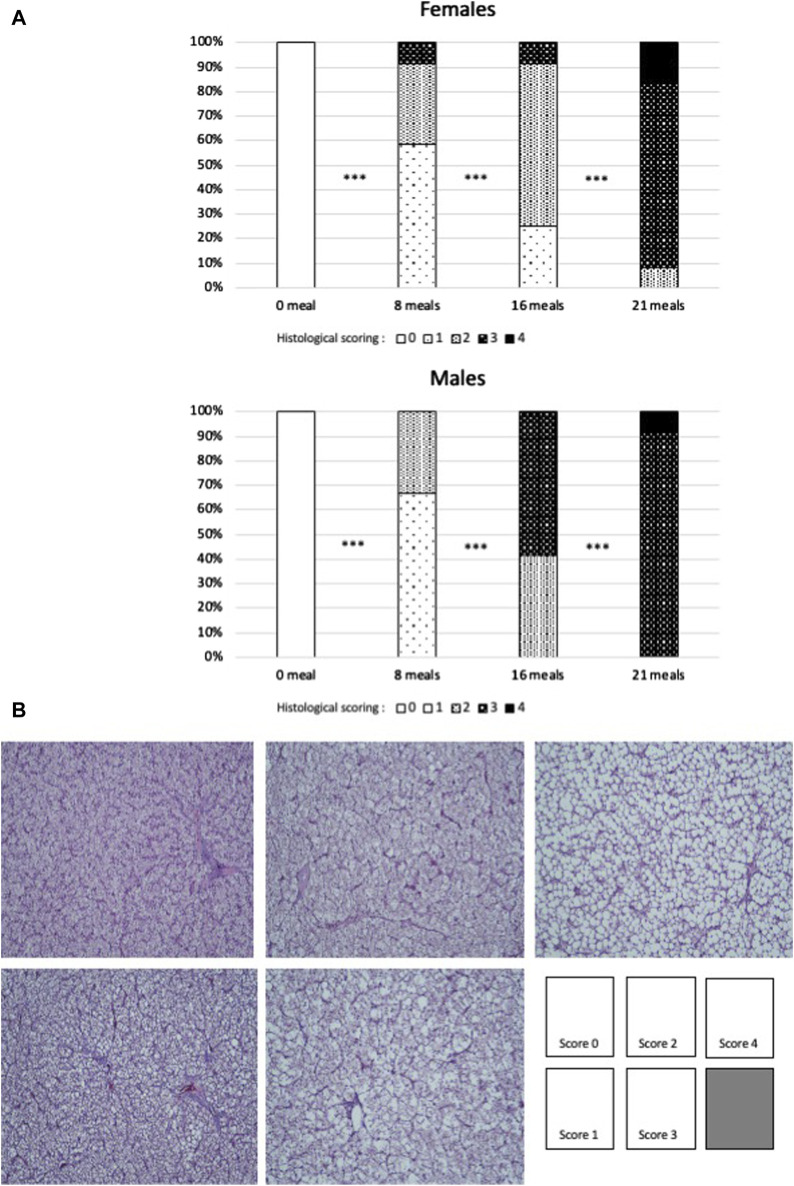
**(A)** Percentages of each score of histological sections of livers of female or male mule ducks according to the number of meals during the force-feeding period. (*n* = 12/sex/stage). Significance between two consecutive stages drawn according to Fisher’s exact test (*** = *p* < 0.001). **(B)** Examples of scoring grades (hematoxylin–eosin staining), with the same magnification for all pictures.

Histological observations (Figure 1) confirm the biochemical measurements regarding lipid accumulation in hepatocytes. In both sexes, lipids were observed to accumulate in hepatocytes in the form of increasingly present and larger droplets throughout the force-feeding period. However, this accumulation seems to be more gradual and less intense in female ducks. At the final stage (after 21 meals of force-feeding), female ducks still presented a low percentage (10%) of hepatocytes with a majority of small droplets (score 2), while male ducks presented only liver cells with a majority of large lipid droplets (score 3 = 90%) or with almost only large lipid droplets (score 4 = 10%). In mice subjected for long periods to unbalanced diets (high-fat, Western, cafeteria, and fructose-enriched diets), male ducks were also found to be more susceptible to hepatic steatosis and had higher histological scores values than female ducks (Spruss et al., 2012; Gasparin et al., 2018; Smati et al., 2022). According to these authors, the prevention of the development hepatic steatosis development in female ducks could be linked to their particular hormonal status, which could also be the case in female ducks submitted to force-feeding (Tramunt et al., 2021).

**FIGURE 2:**
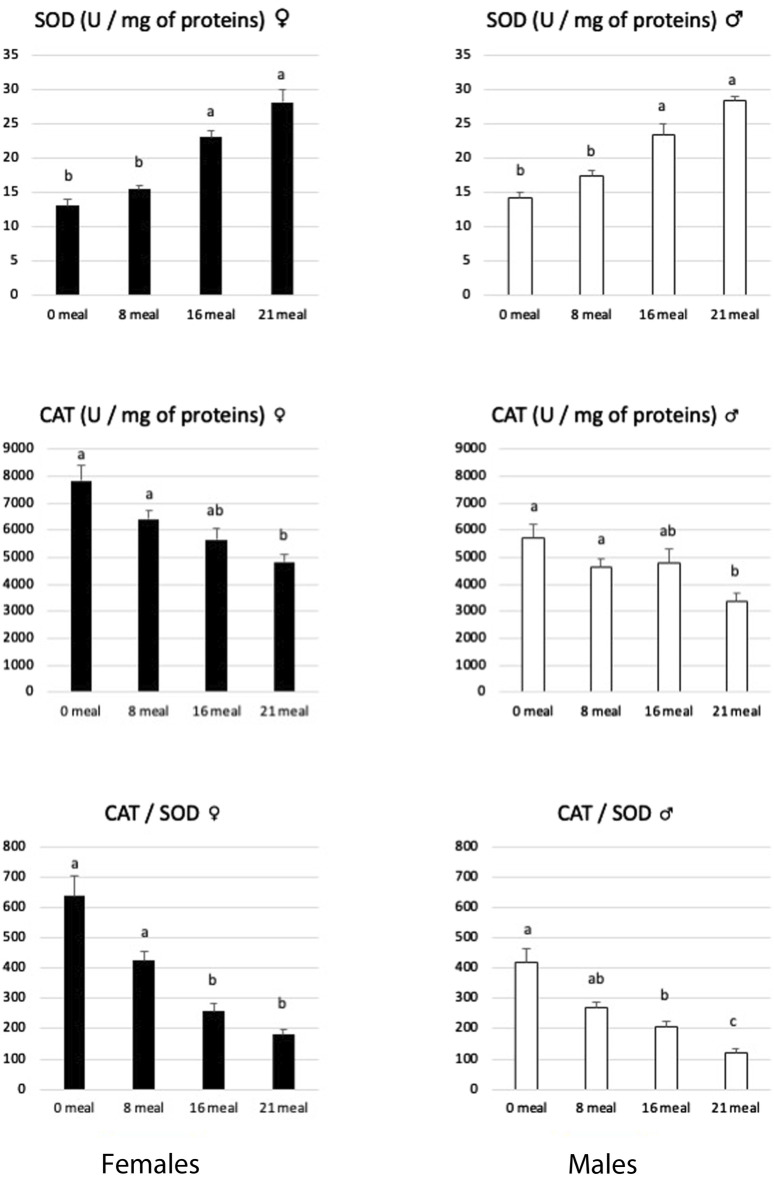
Activities of superoxide dismutase (SOD) and catalase (CAT) enzymes in livers of female and male mule ducks according to the number of meals during the force-feeding period. (*n* = 12/sex/stage). Values are the means ± SD. Values with different superscripts are different (*p* < 0.05).

Corn force-feeding, containing more than 60% starch, induces significant intakes of carbohydrates which mainly arrive in the liver, where they are transformed into lipids. These lipids are initially dedicated to export by lipoproteins (Hermier et al., 2003; Tavernier et al., 2018). However, due to the very large quantities of lipids synthetized, the storage capacity of peripheral tissues, mainly adipose tissues, is quickly exceeded, leading to lipid accumulation in the liver itself. There is therefore an enormous accumulation of complex lipids and free fatty acids in the hepatocytes. This abnormal accumulation of lipids has been described in mammals (Chen et al., 2019) as favoring lipotoxic species such as reactive oxygen species (ROS), which are highly toxic to the cell. However, cells have different mechanisms for neutralizing these ROS, among which the coupling activity of the enzymes superoxide dismutase (SOD) and catalase (Cat) is very effective (Perlemuter et al., 2005) in neutralizing the superoxide ion O_2_^−^, one of the most common ROS. In the present study, SOD activities increased rapidly from eight meals and beyond throughout the force-feeding period in both sexes (Figure 2). On the contrary, Cat activities decrease slightly during the same period in both sexes. An increase in SOD and Cat activities has often been described under different conditions (Zeng et al., 2014; Liu et al., 2015; Du et al., 2017; Zhang et al., 2017; Remignon and Burgues, 2023) to be associated to fight against oxidative stress due to the accumulation of lipids in hepatocytes. However, in mice fed a cafeteria diet, Gasparin et al. (2018) reported decreased Cat activity in the liver and indicated that this suggests impaired H_2_O_2_ neutralization that could contribute to lipid peroxidation. In the present study, this possible accumulation of H_2_O_2_ is highlighted by the decrease in the CAT/SOD ratio (Figure 2) during the force-feeding period in both sexes.

**FIGURE 3:**
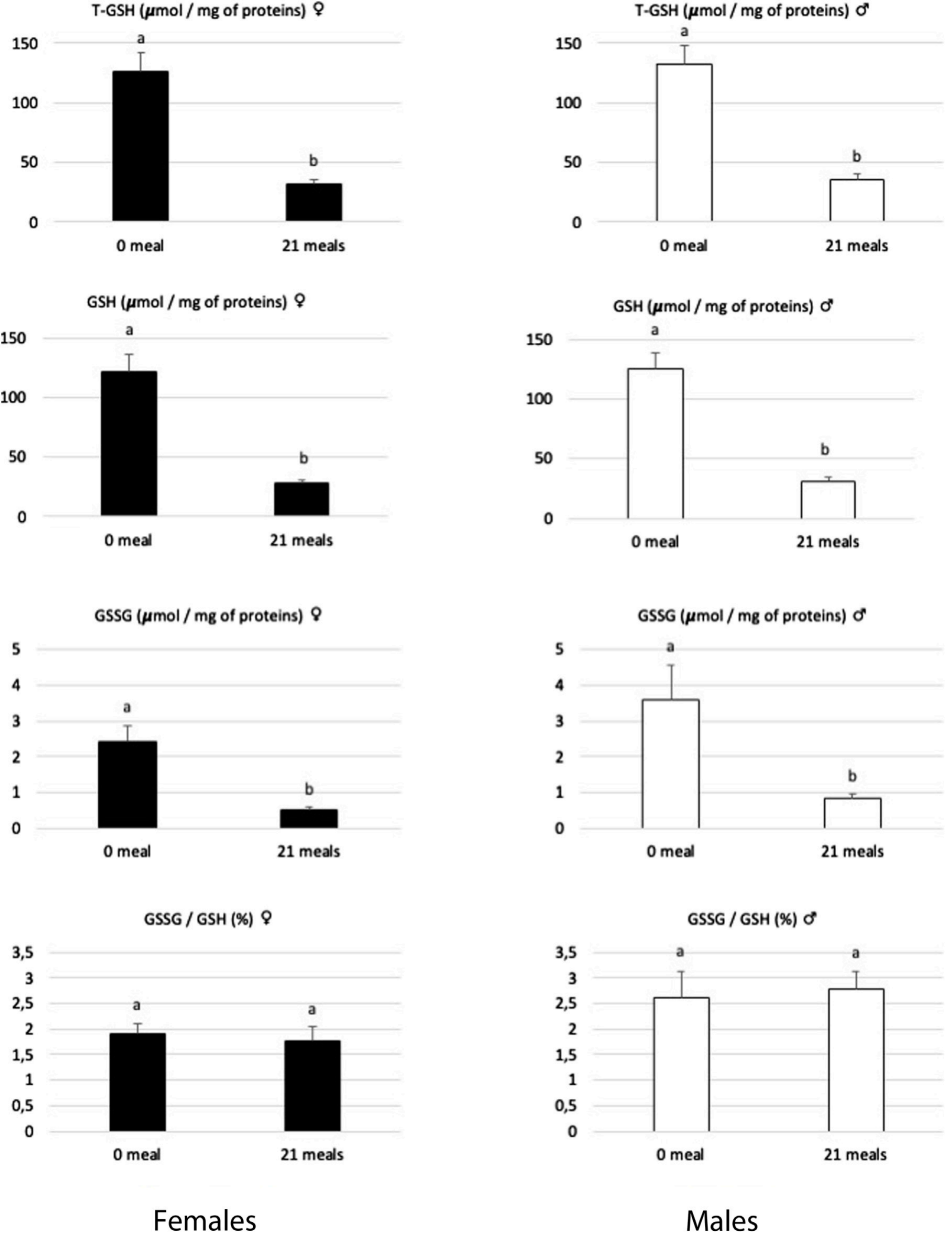
Biochemical contents of total glutathione (T-GSH), reduced glutathione (GSH), oxidized glutathione (GSSG), and the GSSG/GSH ratio in livers of female and male mule ducks before (0 meal, *n* = 10) and after (21 meals, *n* = 10 for T-GSH and *n* = 5 for GSH and GSSG) the force-feeding period. (*n* = 12/sex/stage). Values are the means ± SD. Values with different superscripts are different (SNK, *p* < 0.05).

Other enzymatic antioxidant systems may act to catalyze the reduction of H_2_O_2_ to H_2_O, as is primarily the case of the glutathione system (for review, see Engin, 2017). Reduced glutathione (GSH) has been described (Forman et al., 2009) to play a very effective role in maintaining redox homeostasis and, therefore, protects cells from oxidative damage (Sahoo et al., 2017). Associated with the action of specific peroxidases, the coupling activities of reduced and oxidized glutathione (GSSG) represent the most powerful cellular antioxidant system: the decrease in the GSSG/GSH ratio is therefore considered a major sign of cellular dysfunctions (Chen et al., 2013; Alkazemi et al., 2021). In the present study (Figure 3), the levels of GSH and GSSG in the liver decreased during the force-feeding period in both sexes. However, the GSSG/GSH ratio remained constant during this period, indicating that the renewal mechanisms of this powerful antioxidant continue to be functional. When large quantities of lipids are synthetized rapidly in hepatocytes, as observed during the development of hepatic steatosis, this also induces an increase in the activity of the ß-oxidation pathway and consequently a great leak of electrons which are at the origin of ROS (Svegliati-Baroni et al., 2019; Geng et al., 2021). If produced in large quantities, these ROS will clearly induce mitochondrial dysfunctions (Guo et al., 2013). To cope with this, hepatocytes will activate specific antioxidant mechanisms involving SOD, Cat, and glutathione, but if the ROS level remains high, these defense mechanisms against oxidative stress may prove insufficient, as illustrated by their strong reductions observed in severe syndromes of NAFLD or NASH (Sumida et al., 2013). In the present study, while the activity level of SOD increased, to neutralize superoxide ions, during the force-feeding period in both sexes, the activity level of CAT, GSH, and GSSG contents decreased, indicating that the liver cells of male and female ducks have to face uncontrolled oxidative stress during the rapid development of hepatic steatosis.

**FIGURE 4:**
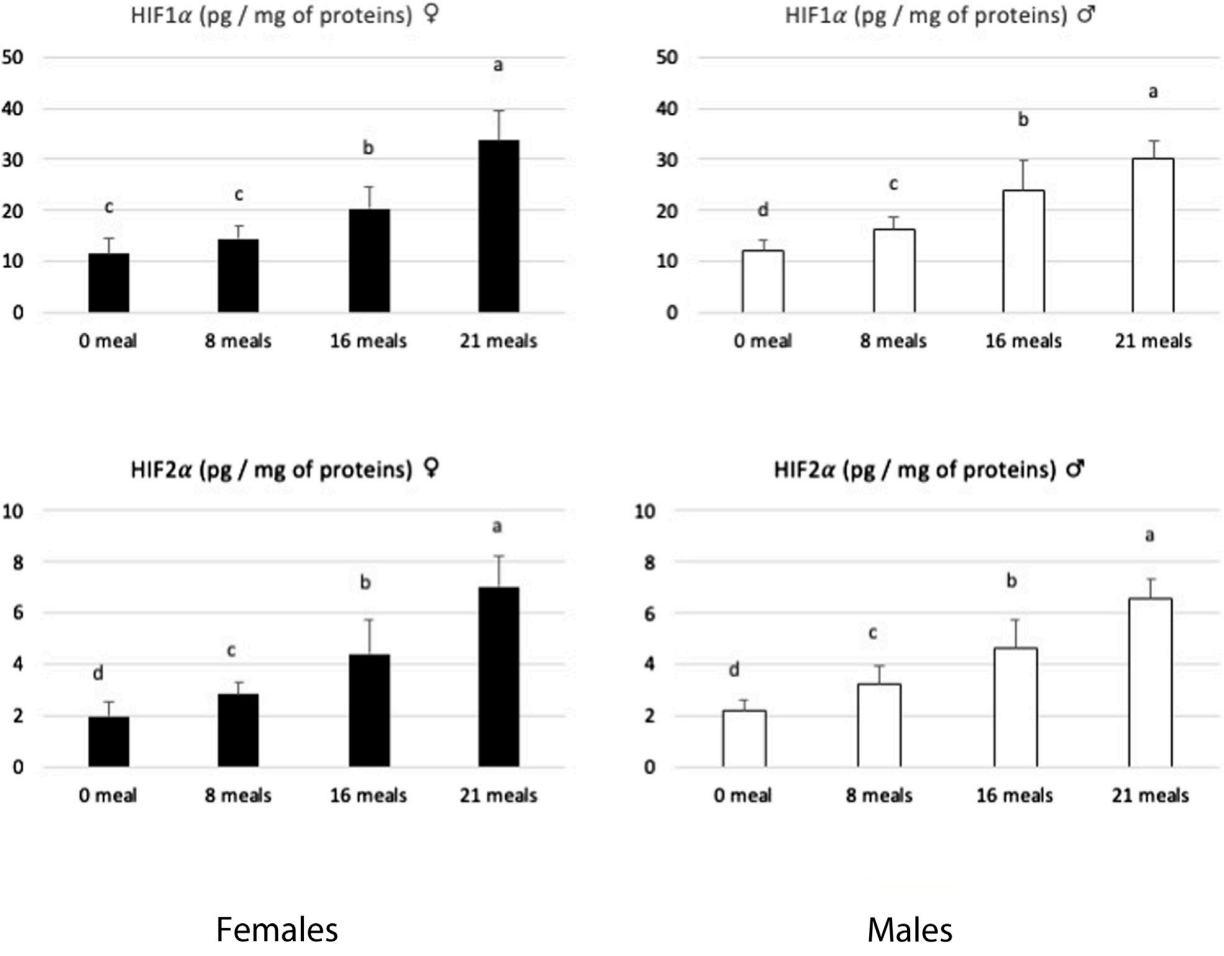
Biochemical contents of hypoxia-inducible factors 1 and 2 (HIF1α and HIF2α) in livers of female and male mule ducks according to the number of meals during the force-feeding period. (*n* = 12/sex/stage). Values are the means ± SD. Values with different superscripts are different (SNK, *p* < 0.05).

The development of hepatic steatosis impairs metabolism and tissue remodeling (Suzuki et al., 2014), mainly through severe hypoxia that disrupts hepatic oxygen homeostasis. This activates the production of transcription factors HIF1α and HIF2α, which are involved in the response to initial or acute hypoxia, respectively (Downes et al., 2018). For example, Morello et al. (2018) reported that HIF2 is overexpressed in humans or mice developing hepatic steatosis of various origins, which leads to a hypoxic microenvironment and mitochondrial dysfunctions. Remignon and Burgues (2023) previously reported an increase in HIF1α and HIF2α factors in the livers of force-fed male mule ducks. In the present study, the evolution of these two hypoxic factors follows the same pattern in male and female ducks (Figure 4). These increases indicate that during the force-feeding period, the livers of both male and female ducks experienced hypoxic conditions that could be related to physical disability due to liver enlargement and/or circulatory problems related to the elevated lipemia typically associated with the development of fatty liver. According to McGarry et al. (2018), an increase in ROS may result in a reduction in the efficiency of oxygen delivery. This could be progressively the case throughout the force-feeding period for both sexes and thus explain the development of the cellular oxidative stress observed independently of any sexual determinism.

According to Medzhitov (2008), inflammation should not be considered solely as a reaction to infection or injury but should be expanded to take into account inflammatory processes induced by other types of adverse conditions. The rapid development of steatosis in the liver, as well as the associated oxidative stress, could therefore induce this type of chronic inflammatory state. TNFα (tumor necrosis factor alpha) and IL18 (interleukin-18) are cytokines which have been shown to be increased in nutritionally induced hepatic steatosis in mice or humans (Henao-Meija et al., 2012; Yamanishi et al., 2016; Tiegs and Horst, 2022; Knorr et al., 2023; Vachliotis and Polyzos, 2023). An increase in the transcription levels of the genes coding for these molecules must therefore be considered a sign of the development of an inflammatory process in the liver. In the present experiment, between the beginning (meal 0) and the end (meal 21) of the force-feeding period, we reported an increase in the transcription level of the TNFα gene in females but not in males and increases in that of IL18 for both sexes (Table 2). This indicates that the inflammatory process was present at the end of the force-feeding period in both sexes, assessed by the increase in the level or transcription of the IL18 gene, but potentially with a higher intensity in females, as illustrated by increases in both levels of transcription IL18 and TNFα genes. This differential level of inflammation in male and female mule ducks during the development of liver steatosis induced by force-feeding has been previously approximated with measurements of OH-Pro levels. On the contrary, Spruss et al. (2012) reported that TNFα levels were altered in male and female mice developing NAFLD.

**TABLE 2:** Fold-changes (references are mean values for 0 meal) of relative expression of selected genes involved in liver metabolisms of male and female mule ducks during the force-feeding period. Values are the means ± SD.

		Gender	0 meal	8 meals	16 meals	21 meals		
	n	Male	9	12	11	10		
		Female	12	9	11	12		
Metabolism	Gene						GIFF*	P^1^<
Carbohydrates	Glut2	Male	1.00 ± 0.08^b^	0.94 ± 0.08^b^	1.40 ± 0.11^a^	1.22 ± 0.15^ab^	**→**	0.0129
		Female	1.00 ± 0.10^a^	0.67 ± 0.04^b^	1.23 ± 0.13^a^	1.19 ± 0.12^a^	**→**	0.0005
	Eno1	Male	1.00 ± 0.04^b^	1.31 ± 0.05^a^	1.25 ± 0.07^a^	1.19 ± 0.13^ab^	↑	0.0261
		Female	1.00 ± 0.07^b^	1.26 ± 0.12^ab^	1.39 ± 0.12^a^	1.42 ± 0.12^a^	↑	0.0219
	Hk1	Male	1.00 ± 0.24^a^	1.2 ± 0.40^a^	1.57 ± 0.40^a^	0.34 ± 0.01^b^	**↓**	0.0060
		Female	1.00 ± 0.34^a^	0.23 ± 0.10^b^	0.83 ± 0.25^a^	0.33 ± 0.08^ab^	**↓**	0.0109
Lipids	Cpt1a	Male	1.00 ± 0.08^b^	0.67 ± 0.05^c^	1.06 ± 0.06^b^	2.51 ± 0.31^a^	**↑**	0.0001
		Female	1.00 ± 0.21^c^	1.86 ± 0.49^b^	1.34 ± 0.10^b^	2.73 ± 0.25^a^	**↑**	0.0001
	Acad11	Male	1.00 ± 0.08^a^	0.39 ± 0.04^b^	0.61 ± 0.03^c^	0.58 ± 0.06^b^	**↓**	0.0001
		Female	1.00 ± 0.07^a^	0.58 ± 0.07^b^	0.60 ± 0.04^b^	0.58 ± 0.04^b^	**↓**	0.0001
	Acox1	Male	1.00 ± 0.06^b^	0.65 ± 0.06^c^	1.05 ± 0.06^b^	1.42 ± 0.17^a^	**↑**	0.0001
		Female	1.00 ± 0.09^b^	0.72 ± 0.08^c^	0.97 ± 0.06^b^	1.45 ± 0.12^a^	**↑**	0.0001
	Soat	Male	1.00 ± 0.11^b^	0.60 ± 0.05^c^	0.77 ± 0.08^b^	1.3 ± 0.17^a^	**↑**	0.0001
		Female	1.00 ± 0.10^b^	0.79 ± 0.07^b^	1.00 ± 0.07^b^	1.53 ± 0.14^a^	**↑**	0.0002
	Dgat2	Male	1.00 ± 0.37	0.63 ± 0.22	1.37 ± 0.43	0.74 ± 0.30	**→**	0.6624
		Female	1.00 ± 0.30	0.58 ± 0.21	0.75 ± 0.25	0.70 ± 0.20	**→**	0.4254
	FasN	Male	1.00 ± 0.06	1.11 ± 0.12	1.02 ± 0.06	0.78 ± 0.08	**→**	0.0653
		Female	1.00 ± 0.13	0.93 ± 0.09	1.24 ± 0.14	1.08 ± 0.11	**→**	0.3841
	Scd1	Male	1.00 ± 0.04^c^	1.60 ± 0.12^b^	1.99 ± 0.11^a^	1.53 ± 0.11^b^	**↑**	0.0001
		Female	1.00 ± 0.15^b^	1.88 ± 0.16^a^	2.43 ± 0.13^a^	2.27 ± 0.25^a^	**↑**	0.0001
	Plin2	Male	1.00 ± 0.11^c^	2.39 ± 0.23^b^	3.87 ± 0.44^a^	2.64 ± 0.14^b^	**↑**	0.0001
		Female	1.00 ± 0.06^b^	0.77 ± 0.11^b^	1.94 ± 0.29^a^	2.18 ± 0.33^a^	**↑**	0.0003
	Fabp4	Male	1.00 ± 0.09^d^	5.00 ± 1.00^c^	49 .00 ± 10.00^b^	121.00 ± 11.00^a^	**↑**	0.0001
		Female	1.00 ± 0.12^d^	5.92 ± 2.11^c^	19.36 ± 2.92^b^	87.30 ± 8.95^a^	**↑**	0.0001
	Apob	Male	1.00 ± 0.40	2.08 ± 0.71	1.03 ± 0.34	1.57 ± 0.45	**→**	0.6743
		Female	1.00 ± 0.20	0.56 ± 0.19	1.00 ± 0.37	0.73 ± 0.28	**→**	0.5012
Inflammation	Il18	Male	1.00 ± 0.17^b^	0.61 ± 0.06^c^	0.90 ± 0.10^b^	1.42 ± 0.14^a^	**↑**	0.0001
		Female	1.00 ± 0.13^c^	1.18 ± 0.11^c^	1.70 ± 0.13^b^	2.68 ± 0.18^a^	**↑**	0.0001
	TNFα	Male	1.00 ± 0.08^a^	0.56 ± 0.04^b^	0.90 ± 0.06^a^	0.96 ± 0.06^a^	→	0.0001
		Female	1.00 ± 0.07^b^	1.00 ± 0.18^b^	1.16 ± 0.09^ab^	1.46 ± 0.13^a^	↑	0.0171
Regulators	Bcl6	Male	1.00 ± 0.16	0.91 ± 0.11	1.30 ± 0.12	1.14 ± 0.11	→	0.0760
		Female	1.00 ± 0.16^b^	0.76 ± 0.09^b^	1.21 ± 0.16^ab^	1.76 ± 0.23^a^	↑	0.0014
	Pparα	Male	1.00 ± 0.04^a^	0.54 ± 0.04^c^	0.76 ± 0.04^b^	0.70 ± 0.06^b^	↓	0.0001
		Female	1.00 ± 0.07	0.88 ± 0.13	1.01 ± 0.07	1.15 ± 0.08	→	0.1214
	Srebp-1c	Male	1.00 ± 0.08^a^	0.61 ± 0.05^b^	0.73 ± 0.09^b^	0.57 ± 0.05^b^	↓	0.0006
		Female	1.00 ± 0.10	0.78 ± 0.17	0.89 ± 0.08	0.75 ± 0.10	→	0.2051
	Chrebp	Male	1.00 ± 0.03^a^	0.60 ± 0.05^b^	0.67 ± 0.04^b^	0.36 ± 0.02^c^	**↓**	0.0001
		Female	1.00 ± 0.07^a^	0.78 ± 0.08^a^	0.81 ± 0.06^a^	0.38 ± 0.04^b^	**↓**	0.0001
	Pparγ	Male	1.00 ± 0.05^a^	0.56 ± 0.04^c^	0.73 ± 0.04^b^	0.71 ± 0.06^b^	↓	0.0001
		Female	1.00 ± 0.06	0.88 ± 0.11	0.95 ± 0.04	1.01 ± 0.06	→	0.3008

Within a line, values with different superscripts are different (*p* < 0.05).

GIFF*: global impact of force-feeding between 0 and 21 meals.

^1^: *p*-values are related to the effect of the number of meals.

BCL6 is a transcription factor that plays a key role in determining the active genetic program in male *versus* female mice and, therefore, their survival under different conditions (Nikkanen et al., 2022). BCL6 is a master transcription factor for the regulation of T follicular helper cells but also acts as a potent antagonist of PPARα-directed gene regulation (Sommars et al., 2019). A high level of hepatic BCL6 in male mice leads to hepatic steatosis and glucose intolerance during dietary excess, while its low level in females may explain better hepatic lipid handling (Salisbury et al., 2021). In the present study, the level of transcription of the BCL6 gene increased only in female ducks during the force-feeding period (Table 2) while it remained constant in males. Since low levels of BCL6 gene transcription in female mice are associated with their increased resistance to the development of hepatic steatosis, we can hypothesize that the observed increase in BCL6 gene transcription in female ducks during the force-feeding period illustrates a decrease in their resistance to hepatic steatosis, which could possibly be linked to their higher inflammatory response. The levels of other transcription factors evaluated (Pparα, Srebp-1c, and Pparγ) decreased in male ducks, while they remained constant in female ducks, during the force-feeding period (Table 2). On the contrary, the transcription level of Chrebp decreased in both sexes. Chrebp (Regnier et al., 2023) and Srebp-1c (Ferré et al., 2021) are involved in lipogenesis pathways when large amounts of glucose are available. In force-fed male ducks, Han et al. (2015) reported that their transcript levels were increased, while we reported them to be downregulated in the present experiment, probably because our birds were fasted for 12 hours before slaughter and, therefore, the level of circulating glucose must have been very low. However, given the enormous amount of lipids accumulated in hepatocytes during force-feeding, this fasting period was not sufficient to increase the level of transcriptional factors such as Pparα or Pparγ, which are generally activated to absorb circulating fatty acids during prolonged periods of fasting (Ruppert and Kersten, 2024).

Other transcript levels regarding carbohydrate or lipid metabolisms in the liver presented changes in male and female ducks during the force-feeding period (Table 2). They are close to previous results (Pioche et al., 2020; Tavernier et al., 2020; Massimino et al., 2021; Andrieux et al., 2023) describing the development of hepatic steatosis induced by force-feeding in male mule ducks. Here, we reported, in male and female mule ducks, that force-feeding influences the transcription of genes (Scd1, Soat, FasN, Dgat2, and Plin2) involved in the neo-synthesis of different types of lipids (fatty acids, polar or neutral lipids, and cholesterol-derived lipids) in response to increasing amounts of carbohydrates delivered. Among the transcripts analyzed and related to lipogenesis, only ACAD11 was significantly decreased during the force-feeding period, as observed by Massimino et al. (2021) in force-fed male mule ducks. For genes related to lipid transport, the relative expression of APOB remained constant, while that of FABP4 was increased significantly during the force-feeding period in both sexes. This has already been observed by Massimino et al. (2021), who proposed two ways to explain these opposing regulations: first, depending on the direction of lipid transport through the liver cells (in or out), their expression can be either increased or decreased by force-feeding; second, the timing of RNA collection after the last meal can have a significant impact on the expression level, as demonstrated by a kinetic study of these same genes by Annabelle et al. (2018). The increase in the level of Fabp4 gene transcripts in the liver is spectacular (121 and 87 times higher in males and females during the entire force-feeding period, respectively), as previously reported in Tavernier et al. (2018) and Pioche et al. (2020). In birds, unlike what is observed in mammals, the liver is the main site of lipid biosynthesis (Cui et al., 2018), and the increase in Fabp4 levels observed during the development of liver steatosis reflects simply the increase in hepatic lipogenesis and transport, as occurring in mammalian adipocytes developing NAFLD (Moreno-Vedia et al., 2022).

## Conclusion

Our experience does not reveal large differences in the independent development of hepatic steatosis in response to specific force-feeding programs adapted for male and female mule ducks. The overall evolution of the different parameters measured was visible in both sexes despite the existence of a proven sexual body dimorphism in lean animals prior to force-feeding. However, female birds could have a higher level of liver inflammation at the end of the force-feeding period. In mammals, we know that females are more resistant than males to developing hepatic steatosis in response to unbalanced diets (Spruss et al., 2012; Gasparin et al., 2018; Smati et al., 2022) but which are incommensurate with those caused by force-feeding of palmipeds. It would therefore be bold to conclude that male and female birds differ from mammals on this point as the modifications induced by force-feeding are particular with regard to the nature of the food, the quantities delivered, and the duration of the force-feeding period.

Our study demonstrates that the natural propensity of palmipeds to rapidly develop hepatic steatosis in response to an energy-rich diet, delivered in large quantities for a very short time, is not specific to males, although only those are traditionally used for the production of foie gras in France. It is better to conclude that despite their domestication, mule ducks, whatever their genus, have retained a particular capacity to produce and store lipids in large quantities in different body compartments. This allows them to support the large energy expenditure initially associated with long migratory flights.

## Data Availability

The datasets presented in this study can be found in online repositories. The names of the repository/repositories and accession number(s) can be found in the article/Supplementary Material.
