# Supplementary material for: Development of hepatic steatosis in male and female mule ducks after respective force-feeding programs
Source: Front Physiol. 2024 Jun 21;15:1392968. doi: 10.3389/fphys.2024.1392968 (PMC11224645; doi:10.3389/fphys.2024.1392968)
Supplement: Supplementary file 1 [file Table1.DOCX]

Supplemental table S1: Informative table on primers used for the study

| **Symbol** | **Name of the gene** | **Forward (5’- 3’)** | **Reverse (3’-5’)** |
| --- | --- | --- | --- |
| GLUT 2 | Glucose transporter type 2 | CGGAGTTGACCAACCCGTTTAT | CTCCACCAGGAACACAGAGATAAC |
| ENO1 | Enolase 1 | GGAATCCCACTGTTGAGGTAGAC | AGCTCTGAACAGACCCTTGTTG |
| HK1 | Hexokinase 1 | TGCCTGGCTACTGCTGCT | GCCGCATGGCGTAAAGGTATT |
| CPT1A | Carnitine palmitoyl transferase 1 | TGGACAGACAAGAAATCAAGCCA | CGGAACAGTTGATCCCATCAGAA |
| PPAR𝛼 | Peroxisome Proliferator-Activated Receptor alpha | AATGGTCCAGGATCTGACGGA | AAGGACTGGATGCTGGTGAAAG |
| ACOX1 | Acyl coenzyme A oxydase 1 | ACCTGGAGATCATTGGCACCTA | AGTTCCATGGCCCATTTCAGTC |
| ACAD11 | Acyl-CoA deshydrogenase | GCAGTGATGTCTCTGTCATTGGA | CCTGCACATGCTGCATTACATAAA |
| BCL6 | B-cell lymphoma 6 | CTCAGCCTTTGGAGAGGAGATG | TCGCAGCTGGAGTCAGAGTAT |
| IL18 | Interleukine 18 | TGGAGAGAACCTCTGCCTCTATT | AGAAGGCATCACATTCCAGCTC |
| TNF𝛼 | Tumor Necrosis Factor alpha | GCCACTGATGTCTTCAATTCCAAA | ATCTTCTTCTGGGCCTGAATGG |
| SOAT1 | Sterol o-acyltransferase | CTCTTCTGCCTGTTTATGTG | GACGGTCGTTAAGAATGAAG |
| SREBP1C | Sterol regulatory element-binding proteins | GCAGAGCAACCAGAAGCTGAA | TCTTCTGCACGGCCATCCT |
| DGAT2 | Diacyglycérol O-acyltranferase 2 | CTGGGCTATATGGAGGTACTTCAG | TGGTCAGCAGATTGTGGGTTT |
| CHREBP | Carbohydrate response element binding protein | TCCTCCACACTGCAAAACTG | ACCATGCCCTTGAAAGACTC |
| SCD1 | Steraoyl-CoA 9 desaturase | ATGCCTGCGCACTTGCTG | ACGGTGGTGGTGCTGGAA |
| FAS | Fatty Acid Synthase | TGGAGTAGCTGCTGTTCAGATTG | CCTGCAGCCTTCAGCATATCA |
| PLIN2 | Perilipin 2 | CGAGTATGCCAGAAAGAACATGAATAG | TCTTCCATTCTACCCAGGATTGATAC |
| PPAR𝞬 | Peroxisome proliferator-activated receptor gamma | CCCAAGTTTGAGTTCGCTGT | GCTGTGACGACTCTGGATGA |
| FABP4 | Fatty acid binding protein 4 | AATGGCTCACTGAAGCAGGT | TGGCTTCTTCATGCCTTTTC |
| APOB | Apolipoprotein | ACCTGCCTGTTATCACCATTCC | TGTATTTGATCCGGCCTTCACTT |
